# Technical advance: The use of tree shrews as a model of pulmonary fibrosis

**DOI:** 10.1371/journal.pone.0241323

**Published:** 2020-11-03

**Authors:** Jennifer L. Larson-Casey, Chao He, Pulin Che, Meimei Wang, Guoqiang Cai, Young-il Kim, Mustapha El Hamdaoui, Rafael Grytz, Qiang Ding, A. Brent Carter

**Affiliations:** 1 Division of Pulmonary, Allergy, and Critical Care Medicine, Department of Medicine, University of Alabama at Birmingham, Birmingham, AL, United States of America; 2 Department of Anesthesiology and Perioperative Medicine, University of Alabama at Birmingham, Birmingham, AL, United States of America; 3 Department of Ophthalmology and Visual Sciences, University of Alabama at Birmingham, Birmingham, AL, United States of America; 4 Birmingham Veteran Affairs Medical Center, Birmingham, AL, United States of America; Medical Center - University of Freiburg, GERMANY

## Abstract

**Background:**

Idiopathic pulmonary fibrosis (IPF) is a chronic, progressive disease with a high morbidity and mortality. Some of the mechanisms of fibrosis development have been described using rodent models; however, the relevance of findings in these animal models is difficult to assess. New innovative models are needed that closely mimic IPF disease pathology.

**Methods:**

To overcome this unmet need of investigating IPF with a relevant model, we utilized tree shrews, which are genetically, anatomically, and metabolically similar to primates and humans. Using human antibodies and primers, we investigated the role of macrophage phenotypic switching in normal and IPF subjects and bleomycin-injured tree shrews.

**Results:**

Bronchoalveolar lavage (BAL) cells from tree shrews expressed human markers, and there was recruitment of monocyte-derived macrophages (MDMs) to the lung in IPF subjects and bleomycin-injured tree shrews. MDMs were polarized to a profibrotic phenotype in IPF and in bleomycin-injured tree shrews. Resident alveolar macrophages (RAMs) expressed proinflammatory markers regardless of bleomycin exposure. Tree shrews developed bleomycin-induced pulmonary fibrosis with architectural distortion in parenchyma and widespread collagen deposition.

**Conclusion:**

The profibrotic polarization of macrophages has been demonstrated to be present in IPF subjects and in fibrotic mice. Although the lung macrophages have long been considered to be homogeneous, recent evidence indicates that these cells are heterogeneous during multiple chronic lung diseases. Here, we show new data that indicate a critical and essential role for macrophage-fibroblast crosstalk promoting fibroblast differentiation and collagen production. in the development and progression of fibrosis. The current data strongly suggest development of therapeutics that attenuate of the profibrotic activation of MDMs may mitigate macrophage-fibroblast interaction. These observations demonstrate that tree shrews are an ideal animal model to investigate the pathogenesis of IPF as they are genetically, anatomically, and metabolically closer to humans than the more commonly used rodent models.

## Introduction

Idiopathic pulmonary fibrosis (IPF) is a chronic, progressive disease that is rapidly increasing in prevalence with over 80 per 100,000 persons developing the disease [1, 2]. The median survival remains to be dismal at 3–5 years [3]. Some of the mechanisms, including alveolar epithelial injury, fibroblast differentiation and extracellular matrix deposition/stiffening, and profibrotic polarization of myeloid cells, have been described using rodent models of fibrosis [4–6]. The relevance of findings in these animal models is difficult to assess due to the dissimilarity of rodents to humans.

We recognize that multiple cell types contribute to IPF development. While existing paradigms have focused on abnormal epithelial-fibroblast interactions in fibrogenesis, a new paradigm based on emerging information from our group and others indicate a critical and essential role for macrophage-fibroblast crosstalk in the development and progression of fibrosis [5, 7, 8]. While macrophages contribute importantly in the immune response to both infectious and non-infectious agents, they are critical in the repair responses to tissue injury [9]. Fibroblasts respond to cytokines and growth factors produced by macrophages to differentiate into myofibroblasts, which generate extracellular matrix (ECM) proteins, possess contractile properties, and invade tissues to mediate repair and remodeling [10, 11].

Macrophage activation is critical in disease development as they can be classically or alternatively activated. These diverse functions are dependent on their ability to polarize between phenotypically distinct sub-populations. Macrophages develop mixed phenotypes in complex pathological conditions, such as lung injury and fibrosis, and polarize to a predominant phenotype depending on the duration and stage of injury repair [8, 12–14]. Alternatively activated, or profibrotic, macrophages express characteristic marker proteins involved in profibrotic changes, such as 1) anti-inflammatory and profibrotic immune effectors, 2) up-regulated L-arginine metabolism by arginase 1 to generate polyamines and proline, and 3) alteration in extracellular matrix dynamics. An imbalance to a predominant profibrotic phenotype can result in a fibrotic response to injury [12, 15].

We and others have shown that lung macrophages from fibrotic mice are profibrotic [5, 7, 8, 16, 17]. Although bronchoalveolar lavage (BAL) cells from IPF subjects are polarized to a profibrotic phenotype, the timing and regulation of this phenotypic switching in fibrosis development/progression is poorly understood. To overcome the unmet need of determining the role of profibrotic polarization of macrophages in humans, we utilized a new animal model, bleomycin-injured tree shrews, to investigate the role of macrophage polarization and macrophage-fibroblast interaction in fibrosis. The beneficial features of this animal model is that tree shrews are genetically, anatomically, and metabolically closer to humans than mice or rats [18–21].

## Materials and methods

### Human subjects

We obtained BAL cells from normal subjects and IPF subjects. Normal subjects had to meet the following criteria: 1) age between 18 and 80 years; 2) no history of cardiopulmonary disease or other chronic disease; 3) no prescription or nonprescription medication except oral contraceptives; 4) no recent or current evidence of infection; and 5) lifetime nonsmoker. IPF subjects had to meet the following criteria: 1) FVC (forced vital capacity) at least 50% predicted; 2) current nonsmoker; 3) no recent or current evidence of infection; and 4) evidence of restrictive physiology on pulmonary function tests and usual interstitial pneumonia on high-resolution chest computed tomography. Fiberoptic bronchoscopy with bronchoalveolar lavage was performed after subjects received local anesthesia. Three sub-segments of the lung were lavaged with five 20-ml aliquots of normal saline, and the first aliquot in each was discarded. The recruitment and bronchoscopy were approved by the University of Alabama at Birmingham and the Birmingham VAMC Institutional Review Boards and conducted between April-December 2019. All subjects were recruited in the UAB or VAMC ILD clinic and provided prior written consent to participate in the study. Subject demographics (Table 1) are representative of a larger population. Table 2 shows forced vital capacity (FVC) measurements for normal and IPF subjects.

**Table 1:** Subject demographics.

	Normal (n = 3)	IPF (n = 5)
**Age, years**	38±9	73±4
**Male sex**	0 (0%)	4 (80%)
**Female sex**	3 (100%)	1 (20%)
**White race**	1 (33%)	5 (100%)
**African American**	2 (67%)	0 (0%)
**Asian**	0 (0%)	0 (0%)
**American Indian/ Alaska Native**	0 (0%)	0 (0%)
**Native Hawaiian or Other Pacific Islander**	0 (0%)	0 (0%)

Demographics of recruited subjects. Data expressed as means ± SD.

**Table 2:** FVC.

	Normal (n = 3)	IPF (n = 5)	p-Value
**FVC (L)**	3.19±0.14	2.48±0.22	0.0027
**FVC (%)**	85.3±15.2	60.2±7.12	0.0170

FVC measurements of recruited subjects. Definition of abbreviation: FVC = forced vital capacity. Data expressed as means ± SD and percent is percent predicted. Independent Student’s *t* test was used to compare means from normal and IPF subjects.

### Tree shrew (*Tupaia belangeri*)

This study was carried out using three- to five-month-old male and female northern tree shrews (*Tupaia Belangeri*), which average cost ranges between $800–1000. Tree shrews were bred in the University of Alabama at Birmingham (UAB) Tree Shrew Core and raised by their mothers until weaning. After weaning, tree shrews were housed in individual cages (1.52m^3^ cage volume) under 14/10 hours light/dark cycles with continuous access to water and dry food. Each cage had a resting board installed. The mating of tree shews can difficult due to their aggressive and territorial nature, but well-matched tree shrews can lead to repeated pregnancies and a successful breeding colony with each litter producing 2–3 tree shrews.

Tree shews were intratracheally administered 1.75 U/kg of bleomycin (*n* = 8) or saline (*n* = 9) after being anesthetized with a mixture of ketamine/xylazine (90 mg/kg Ketamine, 10 mg/kg Xylazine, i.m.) and isoflurane supplement as needed (0.5–2.0%) using a precision Fortec vaporizer. The health of the animals was monitored daily throughout the experiment. Twenty-one days after the intratracheal administration, euthanasia was performed using a lethal injection of Xylazine and bilateral thoracotomy. Bronchoalveolar lavage (BAL) was harvested by cannulating the trachea and performing total lavage of the lungs three times with 7 ml sterile saline using the flow of gravity to collect cells and fluid. All protocols were approved by the University of Alabama at Birmingham Institutional Animal Care and Use Committee, and in accordance with the National Institutes of Health guidelines for animal care.

### Quantitative real time PCR

Total RNA was isolated, reverse transcribed, and quantitative real-time PCR was performed as described previously [7]. The following primer sets were used: human arginase-1: 5’-TTC TCA AAG GGA CAG CCA CG-3’ and 5’-TAG GGA TGT CAG CAA AGG GC-3; human CCL18: 5’-TGC TCC TGT GCA CAA GTT GG-3’ and 5’-CTG GGG GCT GGT TTC AGA AT-3’; human collagen 1α: 5’-GCT CGT GGA AAT GAT GGT GC-3’ and 5’-ACC AGG TTC ACC GCT GTT AC-3’; human fibronectin: 5’-TGT TAT GGA GGA AGC CGA GGT T-3’ and 5’-CGA TGC AGG TAC AGT CCC AGA-3’; human iNOS: 5’-CGG TGC TGT ATT TCC TTA CGA GGC GAA GAA GG-3’ and 5’-GGT GCT GCT TGT TAG GAG GTC AAG TAA AGG GC-3’; human TGF-β1: 5’-CGT GGA GCT GTA CCA GAA ATA C-3’ and 5’-CAC AAC TCC GGT GAC ATC AA-3’; human TNF-α: 5’-CAG CCT CTT CTC CTT CCT GA-3’ and 5’-AGC CTT GGC CCT TGA AGA-3’; human PDGF-B: 5’-AGC CTC GCT GCA AAG AGA AA-3’ and 5’-ATC TTC CTC TCC GGG GTC TC-3; human VEGFA: 5’- CCA TGC AGA TTA TGC GGA TCA AA-3’ and 5’- CAC CAA CGT ACA CGC TCC AG-3’. Data was calculated by the cycle threshold (^ΔΔ^CT) method, normalized to HPRT, and expressed in arbitrary units.

### Flow cytometry

BAL cells were blocked with 1% BSA containing TruStain fcX (anti-human) antibody (422302; BioLegend), followed by staining with antibodies. Antibodies used: LIVE Dead-eflour506 (65–0866; Invitrogen), Rat anti-human CD11b-APC-Cy7 (101226; BioLegend), Mouse anti-human HLA-DR-PE-Cy7 (560651; BD Biosciences), Mouse anti-human CD206-FITC (551135; BD Biosciences), Mouse anti-human CD169-BB700 (745772; BD Biosciences), and Mouse anti-human CD163-PE (560933; BD Biosciences). Hierarchical gating strategy was used to represent the resident alveolar macrophages as CD11b^+^HLA-DR^++^CD206^++^CD169^+^CD163^++^ and monocyte-derived macrophages as CD11b^+^HLA-DR^++^CD206^++^CD169^+^CD163^+^. Data was acquired on ARIA (BD Biosciences) using BD FACS DIVA software (version 8.0.1). Data was analyzed using FlowJo (FlowJo LLC) software (Version 10.5.0).

### IPF fibroblast incubation with BAL fluid

IPF fibroblasts were cultured in DMEM supplemented with 10% fetal bovine serum, streptomycin, and amphotericin B. All experiments were conducted in BAL fluid that was centrifuged to remove debris and cells. IPF fibroblast were incubated in equal concentrations of BAL fluid from tree shrews exposed to saline or bleomycin. IPF fibroblasts were harvested after 24 hr of incubation with BAL fluid.

### Immunoblot analysis

Cell lysates were harvested in lysis buffer containing a protease inhibitor mix (Roche Applied Science, Complete Mini tablets) and a phosphatase inhibitor mix (Calbiochem). Cell lysates were assayed for protein content using a DCTM protein assay kit (Bio-Rad) and separated by SDS-PAGE and transferred to PVDF membranes. Immunoblot analyses on the membranes were performed with the designated antibodies, followed by the appropriate secondary antibody cross-linked to horseradish peroxidase. Primary antibodies used were human β-actin (A5441, Sigma) and human α-SMA (03–61001, American Research Products).

### Hydroxyproline assay

Lung tissue was dried to a stable weight and acid hydrolyzed with 6N HCl for 24 h at 110˚C. Samples were resuspended in 1.5 ml phosphate-buffered saline followed by incubation at 60°C for 1 h. Samples were centrifuged at 13,000 rpm, and the supernatant was taken for hydroxyproline analysis by using chloramine-T. Hydroxyproline concentration was normalized to the dry weight of the tissue.

### Statistics

Statistical comparisons were performed using a student’s t test when only two groups of data are presented, or one-way ANOVA with a Tukey’s post hoc test when multiple data groups are present. All statistical analyses were expressed as ± S.E.M. unless otherwise noted and *p* < 0.05 was considered to be significant. GraphPad Prism 5.0 (GraphPad Software) statistical software was used for all analyses. For comparing 3 normal and 5 IPF subjects, the comparison tests were performed with 80% power to detect 2.06 effect size (smaller mean is 2.06 standard deviation apart from the larger mean) at one-sided α = 0.05. Experiments were performed with 3 to 6 tree shrews per group for two group comparisons with 80% power to detect 1.73 to 2.49 effect sizes at one-sided α = 0.05. For four group comparisons with 3 tree shrews per group at one-sided α = 0.05, the study would be able to detect that difference between the smallest and largest mean is 2.95 standard deviation. As the experiments were performed with expectation of detecting 5 to 15-fold differences of means, utilized number of tree shrews provided sufficient power to observe the group differences.

### Study approval

We obtained BAL cells from normal and IPF subjects under an approved protocols (300001124) by the Human Subjects Institutional Review Board of the University of Alabama at Birmingham and (1670) from the Birmingham Department of Veteran Affairs. Human BAL specimens were used for research only. All subjects provided prior written consent to participate in the study. Animal experiments were approved by the University of Alabama at Birmingham Institutional Animal Care and Use Committee under protocol 21869 and were performed in accordance with NIH guidelines.

## Results

### Recruited monocyte-derived macrophages from tree shrews are increased in fibrosis

**Fig 1:**
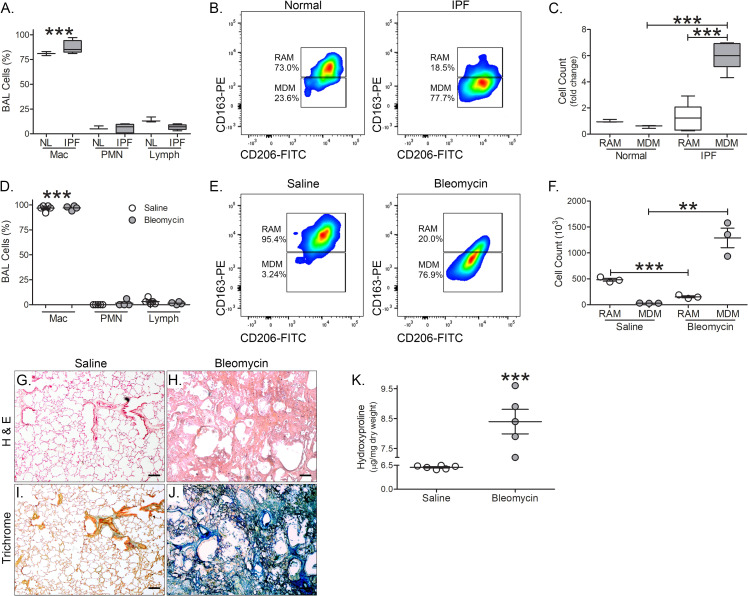
Recruited monocyte-derived macrophages from tree shrews are increased during fibrosis. (A) Cell differential of normal (*n* = 3) and IPF (*n* = 5) subjects. (B) Representative flow cytometry plots of monocyte-derived macrophages (MDM, CD11b^+^HLA-DR^++^CD206^++^CD169^+^CD163^+^) and resident alveolar macrophages (RAM, CD11b^+^HLA-DR^++^CD206^++^CD169^+^CD163^++^) of normal (*n* = 3) and IPF (*n* = 5) subjects. (C) Total cell number of RAM and MDM from BAL of normal (*n* = 3) and IPF (*n* = 5) subjects expressed as fold change. (D) Cell differential of saline- (*n* = 5) and bleomycin-exposed (*n* = 4) tree shrews. (E) Representative flow cytometry plots of monocyte-derived macrophages (MDM, CD11b^+^HLA-DR^++^CD206^++^CD169^+^CD163^+^) and resident alveolar macrophages (RAM, CD11b^+^HLA-DR^++^CD206^++^CD169^+^CD163^++^) of saline- (*n* = 3) and bleomycin-exposed *(n* = 3) tree shrews. (F) Total cell number of RAM and MDM from BAL saline- (*n* = 3) and bleomycin-exposed (*n* = 3) tree shrews. Representative H & E staining of lung tissue from (G) saline- *(n* = 6) and (H) bleomycin-exposed (*n* = 5) tree shews. Masson’s Trichome staining of lung tissue from (I) saline- *(n* = 6) and (J) bleomycin-exposed (*n* = 5) tree shews. Scale bar = 100 μm for G-J. (K) Hydroxyproline analysis of saline- *(n* = 6) and bleomycin-exposed (*n* = 5) tree shrews. **, *p* < 0.001; ***, *p* < 0.0001. Values shown as mean ± S.E.M. Two-way ANOVA with Tukey post-test was utilized for (A), (C), (D), and (F). Two-tailed *t*-test statistical analysis was utilized for (K).

Recent studies indicate that monocyte-derived macrophages (MDMs) are responsible for dysregulated fibrotic repair [17, 22]. Human MDMs and tissue resident alveolar macrophages (RAMs) can be distinguished based on level of expression of CD163 [23]. Macrophages comprise the majority of BAL cells from normal and IPF subjects (Fig 1A). BAL cells were subjected to flow cytometry (S1A Fig). MDMs (CD11b^+^HLA-DR^++^CD206^++^CD169^+^CD163^+^) are the primary cell type in BAL fluid from IPF subjects compared to normal subjects (Fig 1B and 1C); however, RAMs (CD11b^+^HLA-DR^++^CD206^++^CD169^+^CD163^++^) are more prevalent in normal subjects compared with IPF subjects.

BAL cells from tree shrews had greater than 90% being monocytic regardless of bleomycin exposure (Fig 1D). Using the same human antibodies and flow strategy (S1B Fig), we found that bleomycin-injured tree shrews had significantly more MDMs than the saline controls (Fig 1E and 1F). Because MDMs are increased in the BAL after bleomycin injury, we determined if bleomycin resulted in fibrosis development in the lungs of tree shrews similar to rodents. The tree shrews had normal lung parenchyma (Fig 1G), whereas there was a dramatic increase in cellular infiltrate and distortion of parenchymal architecture after bleomycin (Fig 1H). Lung histology from bleomycin-injured tree shrews was similar to previous findings from bleomycin-exposed mice. Collagen deposition was widespread in bleomycin-injured animals compared to the saline controls (Fig 1I and 1J). The histological findings were verified biochemically by hydroxyproline analysis (Fig 1K). In aggregate, these observations suggest that macrophages from tree shrews express human markers, there is recruitment of MDMs to the lung after bleomycin injury, and tree shrews develop bleomycin-induced pulmonary fibrosis.

### Recruited monocyte-derived macrophages are polarized to a profibrotic phenotype

**Fig 2:**
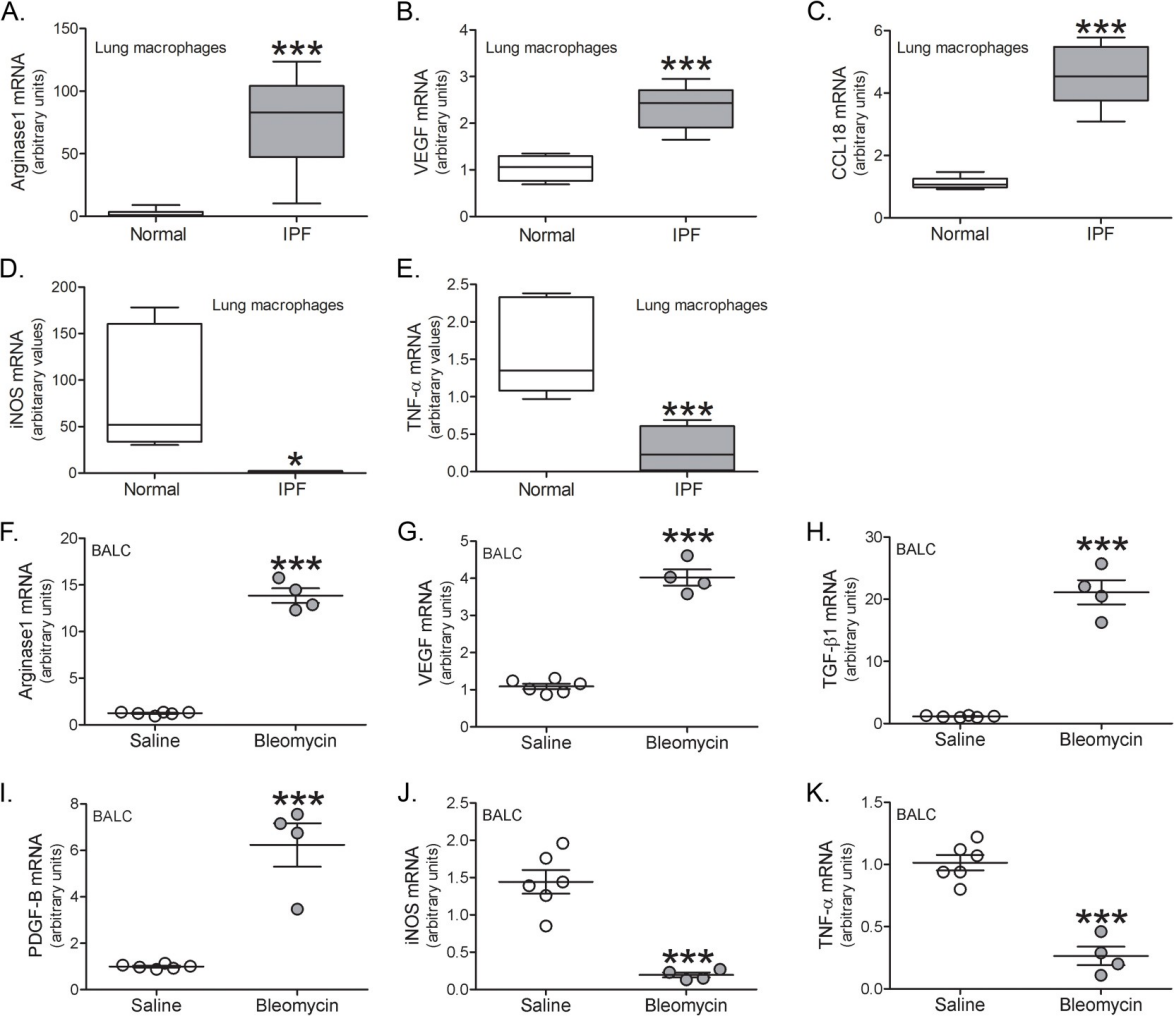
BAL cells are polarized to a profibrotic phenotype. (A) Arginase 1, (B) VEGF, (C) CCL18, (D) iNOS, and (E) TNF-α mRNA expression in BAL cells from normal (*n* = 5–7) or IPF subjects (*n* = 5–6). BAL cells were isolated and mRNA expression was determined from saline- (*n* = 6) and bleomycin-exposed (*n* = 4) tree shrews for (F) arginase 1, (G) VEGF, (H) TGF-β, (I) PDGF-B, (J) iNOS, and (K) TNF-α. *, *p* < 0.01; ***, *p* < 0.0001. Values shown as mean ± S.E.M. Two-tailed *t*-test statistical analysis was utilized.

We and others have shown that BAL cells from subjects with pulmonary fibrosis and fibrotic mice have a profibrotic phenotype [5, 8, 12, 16, 24]. We questioned if BAL cells from IPF subjects expressed profibrotic genes compared to normal subjects. BAL macrophages from IPF subjects expressed greater arginase 1 (Fig 2A), VEGF (Fig 2B), and CCL18 (Fig 2C). In contrast, proinflammatory genes, iNOS and TNF-α, were significantly greater in the normal subjects (Fig 2D and 2E).

A similar polarization of macrophages was observed in tree shrews after bleomycin injury. Using primers for human genes, arginase 1 (Fig 2F), VEGF (Fig 2G), TGF-β1 (Fig 2H), and PDGF-B (Fig 2I) were increased in lung macrophages from bleomycin-injured tree shrews. The proinflammatory markers, iNOS (Fig 2J) and TNF-α (Fig 2K), were decreased after bleomycin.

**Fig 3:**
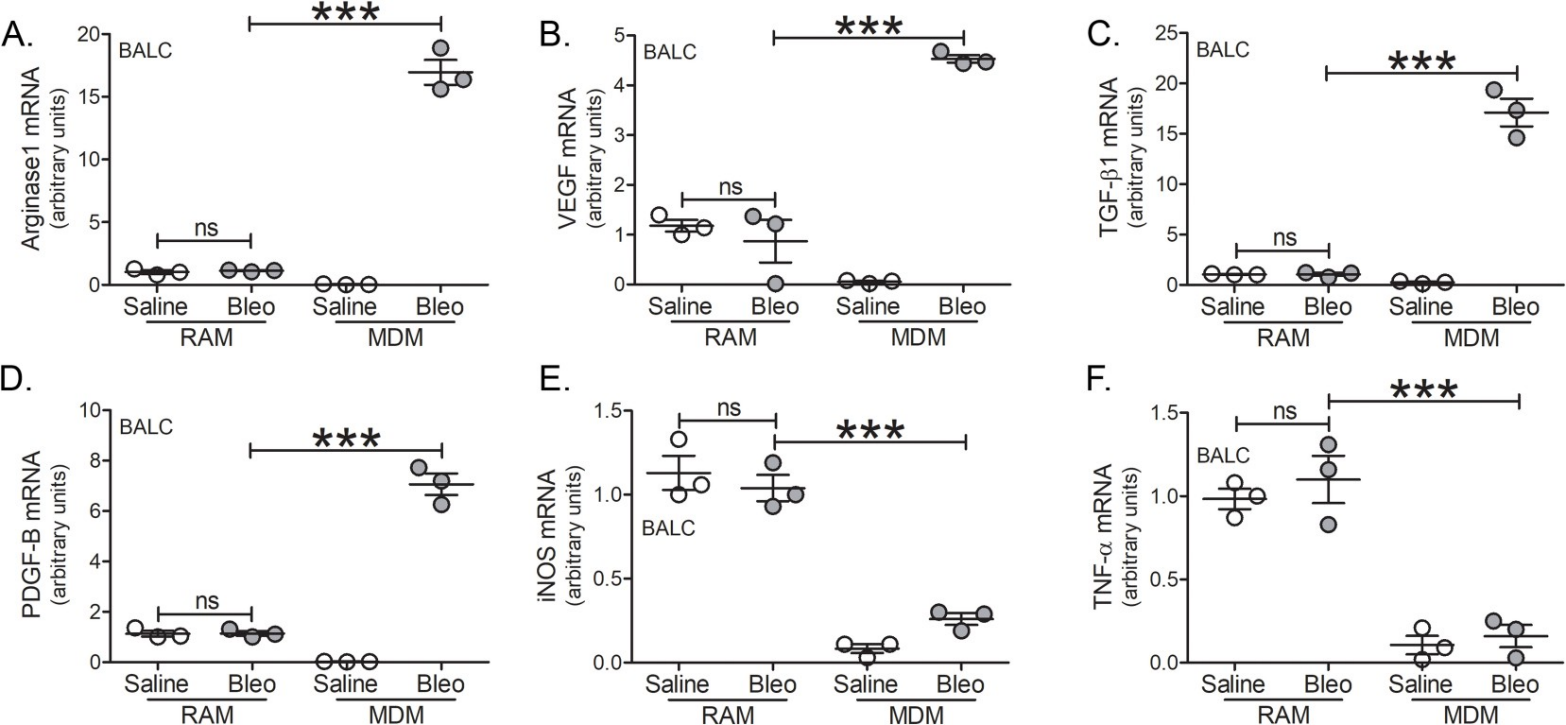
Recruited monocyte-derived macrophages are polarized to a profibrotic phenotype. Tree shrews were exposed to saline (*n* = 3) or bleomycin (*n* = 3) and BAL cells were isolated. FACS-sorted RAMs and MDMs were analyzed for (A) arginase 1, (B) VEGF, (C) TGF-β, (D) PDGF-B, (E) iNOS, and (F) TNF-α mRNA expression. ***, *p* < 0.0001. Values shown as mean ± S.E.M. One-way ANOVA with Tukey post-test was utilized.

To determine the macrophage subset that polarized to the profibrotic phenotype, BAL cells were subjected to FACS sorting to separate RAMs and MDMs. All of the profibrotic markers, arginase 1 (Fig 3A), VEGF (Fig 3B), TGF-β1 (Fig 3C), and PDGF-B (Fig 3D) were significantly greater in MDMs than RAMs from bleomycin-injured tree shrews. The gene expression of iNOS (Fig 3E) and TNF-α (Fig 3F) were reduced in MDMs and no different from the MDMs from the saline controls. The reverse was seen with the RAMs as they had a proinflammatory phenotype regardless of bleomycin. Taken together, these data demonstrate that the profibrotic polarization of lung macrophages occurs exclusively in MDMs and supports the fact that their recruitment is necessary for dysregulated repair in pulmonary fibrosis. Furthermore, tree shrews represent a novel model to investigate IPF since these animals are genetically and immunologically similar to humans.

### Macrophage-derived profibrotic gene expression regulates fibroblast differentiation

**Fig 4:**
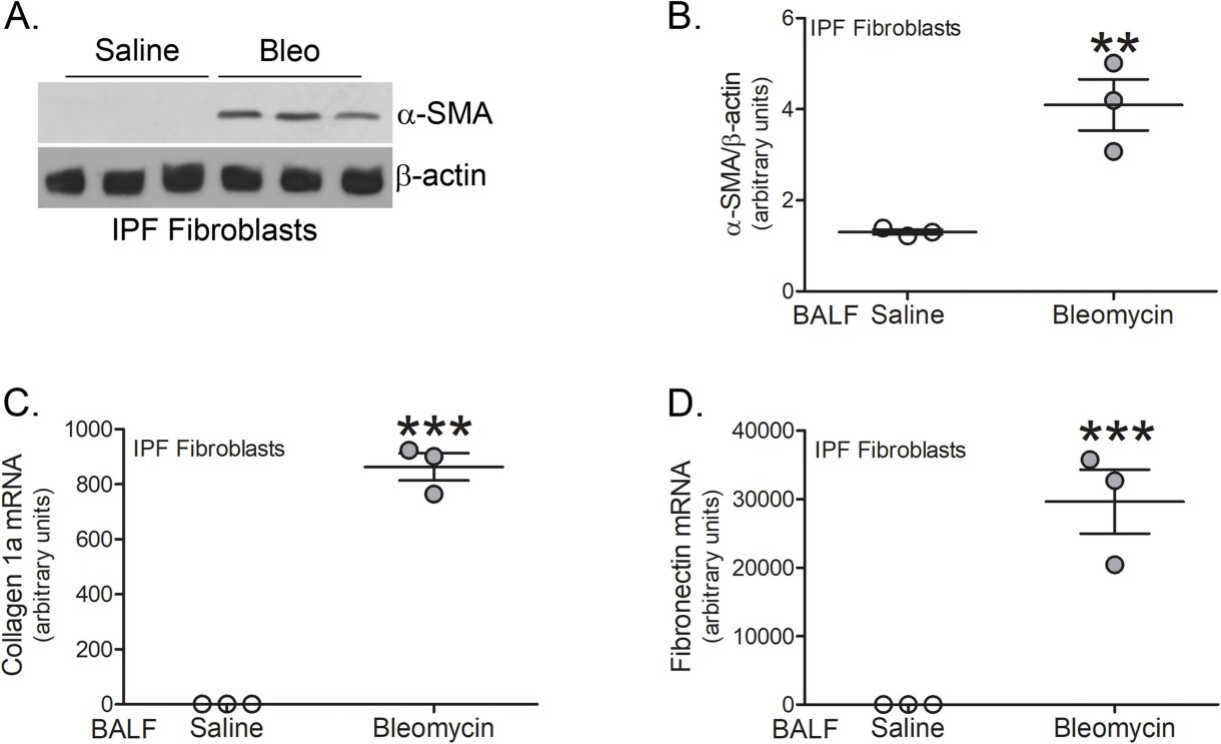
Macrophage-derived profibrotic gene expression regulates fibroblast differentiation. (A) Immunoblot analysis of IPF fibroblasts cultured in BAL fluid from saline- (*n* = 3) or bleomycin-exposed (*n* = 3) tree shrews. (B) Quantification of α-SMA expression in A (*n* = 3 per group). mRNA analysis of (C) collagen 1a and (D) fibronectin in IPF fibroblasts cultured in BAL fluid from saline- (*n* = 3) or bleomycin-exposed (*n* = 3) tree shrews. **, *p* < 0.001; ***, *p* < 0.0001. Values shown as mean ± S.E.M. Two-tailed *t*-test statistical analysis was utilized.

We have shown that active TGF-β1 in BAL fluid is primarily derived from lung macrophages and is essential for fibrosis as *Tgfb1*^*-/-*^*Lyz2-cre* mice are protected from fibrosis development [7]. To determine if BAL fluid from tree shrews could regulate fibroblast differentiation and ECM gene expression, we incubated IPF fibroblasts with the BAL fluid from tree shrews. An immunoblot analysis revealed that BAL fluid from bleomycin-injured tree shrews greatly increased α-SMA protein in IPF fibroblasts compared to the saline controls (Fig 4A and 4B). The expression of collagen 1a (Fig 4C) and fibronectin (Fig 4D) was also significantly increased by culturing IPF fibroblasts in BAL fluid from bleomycin-injured tree shrews. These observations suggest that the macrophage-derived profibrotic mediators from bleomycin-injured tree shrews promote fibroblast activation.

## Discussion

Fibrotic remodeling affects all organ systems, including the heart, kidney, liver, vasculature, and lung. It is estimated that 45% of deaths in the United States are accredited to fibrotic disease annually [25]. IPF, in particular, carries a high mortality with a median survival rate of less than three years, and only 20% of patients survive 5 years after diagnosis [26]. The incidence and mortality from IPF is increasing worldwide with an estimated 40,000 lives lost in the U.S. each year [2, 27]. Due to the extreme morbidity and mortality of IPF, there is a critical and unmet need for a more innovative model that will facilitate the study of IPF and further the development and testing of novel therapeutics to treat this devastating disease.

Although the lung macrophages have long been considered to be homogeneous, recent evidence indicates that these cells are heterogeneous during multiple chronic lung diseases [5, 17, 28–30]. Importantly, the critical role of recruited monocyte-derived macrophages is known to be necessary for the development of fibrosis [5, 17, 22]; however, little is known about the subset of macrophages that are profibrotic, which is essential for the dysregulated fibrotic repair. The profibrotic polarization of macrophages has been demonstrated to be present in IPF subjects and in bleomycin-injured and asbestos-exposed fibrotic mice [5, 7, 8, 16, 17]. The presence of proinflammatory macrophages in IPF is much different. For example, conflicting data exists on the expression of iNOS in humans [31, 32]; however, little is known about the expression in IPF. Our data shows that iNOS is significantly decreased in macrophages from IPF subjects, and a similar trend was seen in bleomycin-injured tree shrew macrophages. We further determined that expression is subset specific. MDMs had significantly reduced iNOS expression compared to RAMs from bleomycin-injured tree shrews. In aggregate, our data demonstrate that MDMs are the macrophage subset that polarized to a profibrotic phenotype in bleomycin-injured tree shrews.

While there are clear advantages to using mice, including inbred strains, large litter size, availability of antibodies, and ease of developing transgenic/knockout mice, tree shrews are an innovative model to study human diseases because they are anatomically and genetically similar to humans [18, 20, 21]. Although non-human primates may be the most human-like model, several impracticalities exist including long reproductive cycle, large animal size, high cost, and slow aging process. Tree shrews present an unique opportunity for translational pulmonary research as they are small animals (150–220 grams), have a low cost of maintenance, short reproductive cycle (41–55 days), and attain sexual maturity at 3–6 months [18]. Ophthalmologic studies are one area in which tree shrews are a valuable model because myopia and collagen remodeling of the sclera are similar to the human disease [33, 34]. In regard to lung disease, tree shrews have been used for lung cancer [35], Zika viral infection [36], and H1N1 influenza infection [37]. The influenza infection in tree shrews is physiologically analogous to the infection in humans.

While the immune system in the mouse greatly differs from that of humans, tree shrews possess the same ligand families as humans, including the major histocompatibility complex (MHC) class I-related chain (MIC) gene and the UL16-binding protein (ULBP) family [18, 38]. Anatomically, the tree shrew lung is more similar to humans than mice. Despite the advantages of the tree shrew as an animal model, limited access to this animal and lack of specific antibodies/reagents restrict the usefulness of this model. A limitation of our study is that we did not evaluate the self-resolving nature of bleomycin-induced lung injury in our tree shrews, as our experiments analyzed fibrosis 21 days after exposure. Additionally, the current study did not assess the ability of the tree shrew model to recapitulate the pathological features of human IPF. Nonetheless, the utilization of human antibodies and primers for the first time in an animal model of pulmonary fibrosis strongly suggest that tree shrews are an ideal model to investigate dysregulated fibrotic repair.

The current dogma of the pathogenesis of pulmonary fibrosis has focused on abnormal epithelial-fibroblast interactions [39–41]. Here, we show observations that indicate a critical and essential role for monocyte-derived macrophage-fibroblast crosstalk in the development and progression of fibrosis. Fibroblasts are the final effector cells in fibrosis. We have demonstrated that the growth factors that induce fibrosis by regulating fibroblast activation are macrophage-derived [7]. More specifically, mice with a conditional deletion of *Tgfb1* in macrophages were protected from pulmonary fibrosis. The current data strongly suggest that attenuation of the profibrotic activation of MDMs mitigates macrophage-fibroblast interaction. Thus, we propose that monocyte-derived macrophage-fibroblast crosstalk can induce fibrosis and is the primary driver in mediating disease progression. Taken together, these observations demonstrate that tree shrews are an ideal animal model to investigate the pathogenesis of IPF as they are genetically, anatomically, and metabolically closer to humans than the more commonly used rodent models.

## Supporting information

S1 Checklist ARRIVE guideline checklist. (DOCX)

S1 Fig Hierarchical gating strategy used to represent resident alveolar macrophages and monocyte-derived macrophages. (TIF)

S2 Fig Uncropped immunoblots. (TIF)
