# Supplementary material for: Technical advance: The use of tree shrews as a model of pulmonary fibrosis
Source: PLoS One. 2020 Nov 3;15(11):e0241323. doi: 10.1371/journal.pone.0241323 (PMC7608928; doi:10.1371/journal.pone.0241323)
Supplement: S1 Checklist — (DOCX) [file pone.0241323.s001.docx]

ARRIVE Guidelines Checklist

1. Study design: This study was carried out using three- to five-month-old male and female northern tree shrews (Tupaia Belangeri). Tree shrews were bred in the University of Alabama at Birmingham (UAB) Tree Shrew Core and raised by their mothers until weaning. After weaning, tree shrews were housed in individual cages (1.52m^3^ cage volume) under 14/10 hours light/dark cycles with continuous access to water and dry food. Each cage had a resting board installed. Tree shews were intratracheally administered 1.75 U/kg of bleomycin (*n* = 8) or saline (*n* = 9) after being anesthetized with a mixture of ketamine/xylazine (90 mg/kg Ketamine, 10 mg/kg Xylazine, i.m.) and isoflurane supplement as needed (0.5-2.0%). The health of the animals was monitored daily throughout the experiment.
2. Sample size: To perform comparison tests with 80% power to detect 2.06 effect size (smaller mean is 2.06 standard deviation apart from the larger mean) at one-sided α=0.05, we utilized 3 normal and 5 IPF subjects. Experiments were performed with 3 to 6 tree shrews per group for two group comparisons with 80% power to detect 1.73 to 2.49 effect sizes at one-sided α=0.05. For four group comparisons with 3 tree shrews per group at one-sided α=0.05, the study would be able to detect that difference between the smallest and largest mean is 2.95 standard deviation. As the experiments were performed with expectation of detecting 5 to 15-fold differences of means, utilized number of tree shrews provided sufficient power to observe the group differences.
3. Inclusion and exclusion criteria: The animals were included in this study if they underwent successful intratracheal injection, defined by visual confirmation of insertion into the trachea. The animals were excluded if insertion of the flexible tipped syringe into the trachea could not be visually confirmed or if the animal died prematurely, preventing collection of BAL.
4. Randomization: Three- to five-month-old male and female northern tree shrews (*Tupaia Belangeri*), weighing 150-220 g, were bred in the UAB Tree Shrew Core and randomly divided into two groups: saline or bleomycin exposed groups.
5. Blinding: For each animal, three different investigators were involved as follows: a first investigator was responsible for the anesthetic procedure. A second investigator administered the exposure to saline or bleomycin and performed the BAL. A third investigator surgically removed the lungs assessed lung histology and hydroxyproline analysis.
6. Outcome measures: This study assessed: development of pulmonary fibrosis, determined by lung histology and hydroxyproline analysis, recruitment of monocyte-derived macrophages, determined by flow cytometry approaches, and expression of profibrotic mediators, determined by mRNA analysis.
7. Statistical methods: Statistical comparisons were performed using a student’s t test when only two groups of data are presented, or one-way ANOVA with a Tukey’s post hoc test when multiple data groups are present. All statistical analyses were expressed as ±S.E.M. unless otherwise noted and *p* < 0.05 was considered to be significant. GraphPad Prism 5.0 (GraphPad Software) statistical software was used for all analyses.
8. Experimental animals: Animals were obtained from the tree shrew colony of the University of Alabama at Birmingham. Animals were housed individually in 1.52 m^3^ cages under 14/10 hours light/dark cycles with continuous access to a water bottles and dry food. Each cage had a resting board installed. The health of the animal was monitored on a daily basis. Male and female three- to five-month-old northern tree shrews (*Tupaia Belangeri*) were utilized for this study.
9. Experimental procedures: Detailed information of experimental procedures is included in the methods section.
10. Results: All statistical analyses were expressed as ±S.E.M. unless otherwise noted and *p* < 0.05 was considered to be significant. GraphPad Prism 5.0 (GraphPad Software) statistical software was used for all analyses. Statistical comparisons were performed using a student’s t test when only two groups of data are presented, or one-way ANOVA with a Tukey’s post hoc test when multiple data groups are present. A summary of the statistics used for each experiment is described in the legend for each figure or table.
