# Supplementary figures and images for: Technical advance: The use of tree shrews as a model of pulmonary fibrosis
Source: PLoS One. 2020 Nov 3;15(11):e0241323. doi: 10.1371/journal.pone.0241323 (PMC7608928; doi:10.1371/journal.pone.0241323)

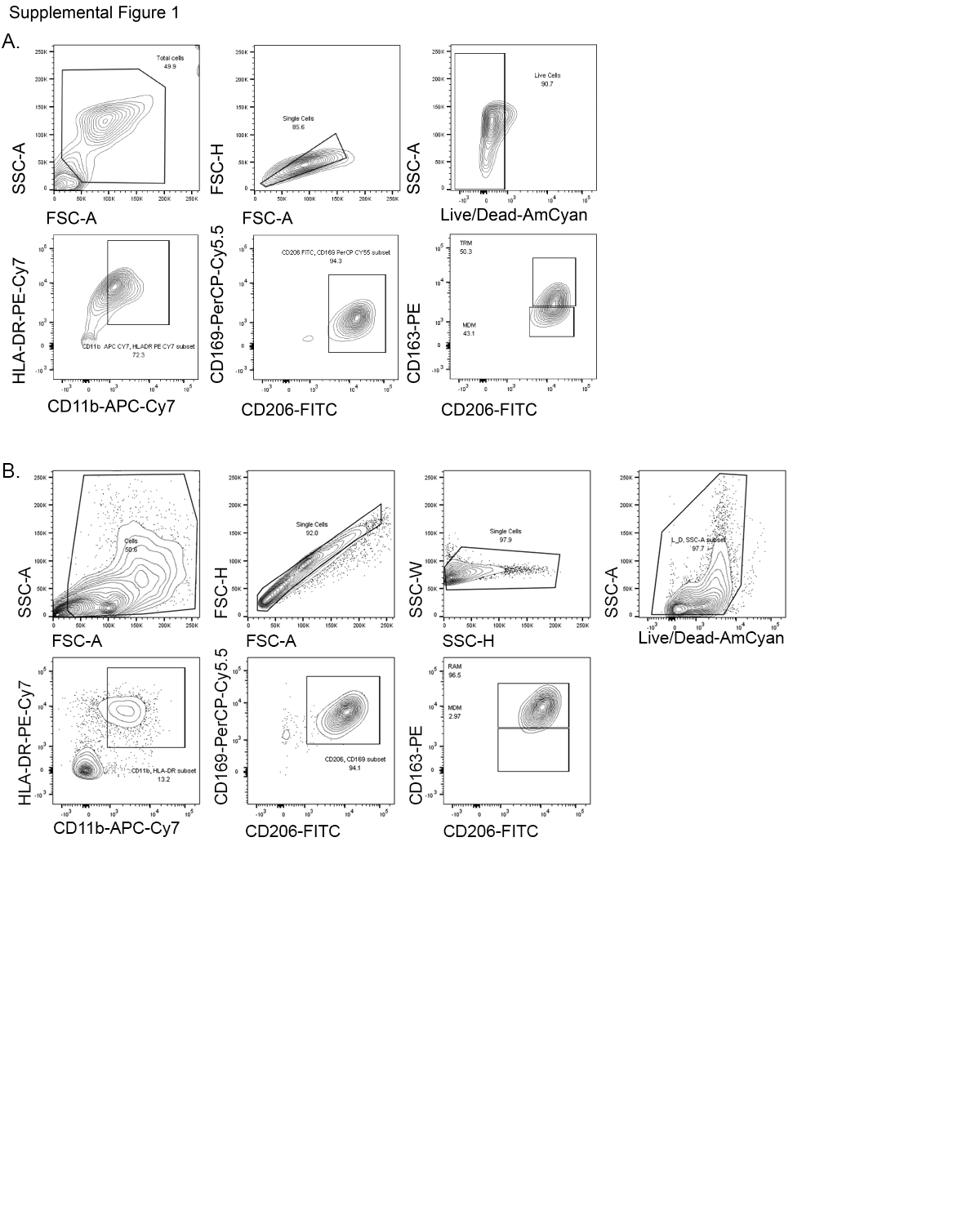

Supplement: S1 Fig — (TIF) [file pone.0241323.s002.tif]

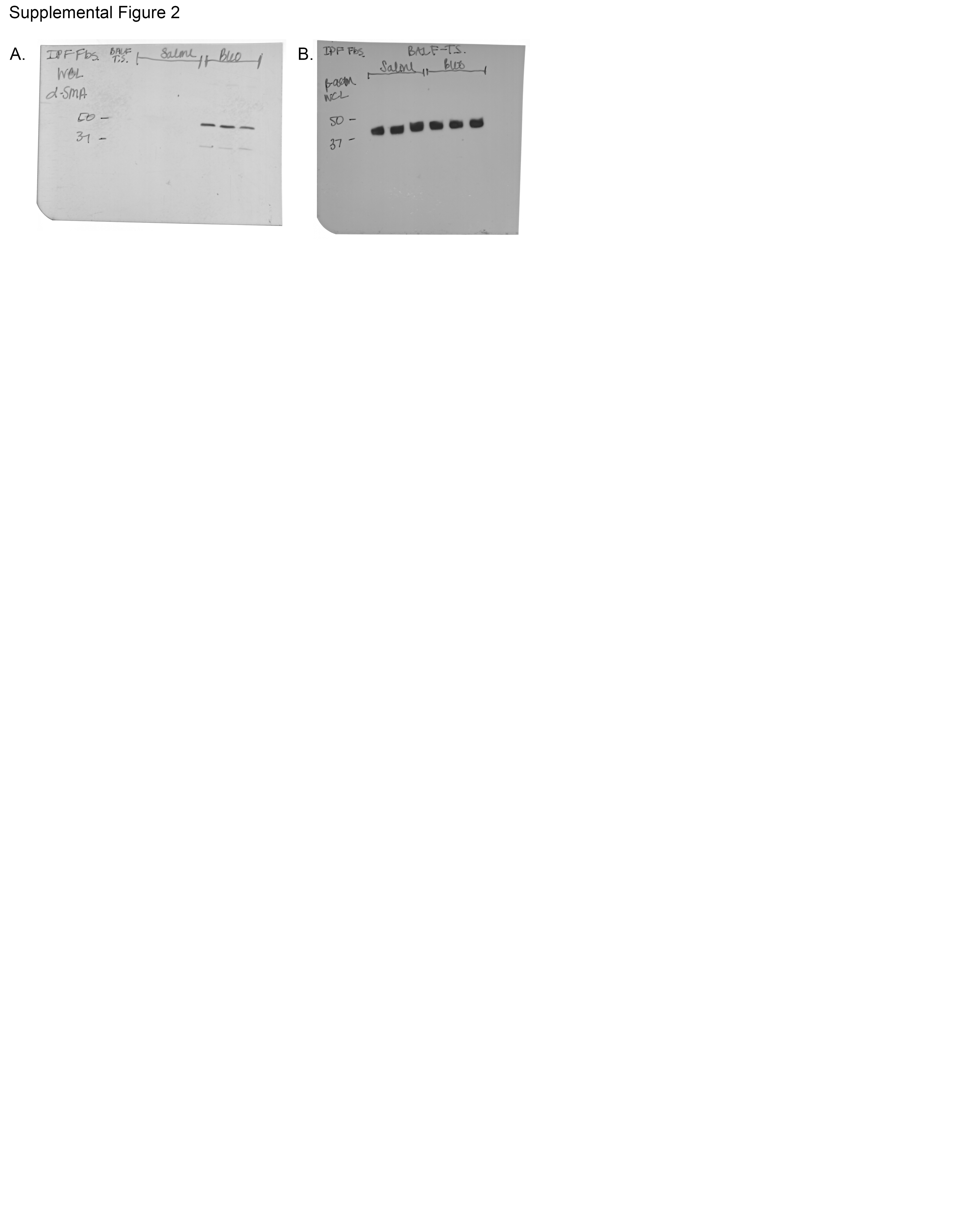

Supplement: S2 Fig — (TIF) [file pone.0241323.s003.tif]
